# Dopamine D2/D3 receptor abnormalities after traumatic brain injury and their relationship to post-traumatic depression

**DOI:** 10.1016/j.nicl.2019.101950

**Published:** 2019-07-22

**Authors:** Amy E. Jolly, Vanessa Raymont, James H. Cole, Alex Whittington, Gregory Scott, Sara De Simoni, Graham Searle, Roger N. Gunn, David J. Sharp

**Affiliations:** a Division of Brain Sciences, Department of Medicine, Imperial College London, UK; b Centre of Dementia Prevention, Centre for Clinical Brain Sciences, University of Edinburgh, UK; c Invicro, Centre for Imaging Sciences, Imperial College London, UK; d Department of Psychiatry, University of Oxford, UK

**Keywords:** Traumatic brain injury, Dopamine, Depression, PET

## Abstract

**Objective:**

To investigate dopamine D2/D3 receptor availability following traumatic brain injury (TBI) and their relationship to the presence of DSM-IV Major Depressive Disorder (MDD) and patterns of axonal injury.

**Methods:**

Twelve moderate-severe TBI patients and 26 controls were imaged using [^11^C]PHNO positron emission tomography (PET) and structural magnetic resonance imaging (MRI). TBI patients and a second group of 32 controls also underwent diffusion tensor imaging (DTI) and neuropsychological assessment. Patients included six with post-injury MDD (TBI-MDD) and six without (TBI-NON). Non-displaceable binding potential (BP_ND_) [^11^C]PHNO values were used to index D2/D3 receptor availability, and were calculated using a reference region procedure. Differences in BP_ND_ were examined using voxelwise and region-of-interest analyses. White matter microstructure integrity, quantified by fractional anisotropy (FA), was assessed and correlated with BP_ND_.

**Results:**

Lower [^11^C]PHNO BP_ND_ was found in the caudate across all TBI patients when compared to controls. Lower [^11^C]PHNO BP_ND_ was observed in the caudate of TBI-MDD patients and increased [^11^C]PHNO BP_ND_ in the Amygdala of TBI-NON patients compared to controls. There were no significant differences in [^11^C]PHNO BP_ND_ between TBI-MDD and TBI-NON patients. Furthermore, DTI provided evidence of axonal injury following TBI. The uncinate fasciculus and cingulum had abnormally low FA, with the uncinate particularly affected in TBI-MDD patients. Caudate [^11^C]PHNO BP_ND_ correlated with FA within the nigro-caudate tract.

**Conclusions:**

[^11^C]PHNO BP_ND_ is abnormal following TBI, which indicates post-traumatic changes in D2/D3 receptors. Patterns of [^11^C]PHNO BP_ND_ seen in patients with and without MDD suggest that further research would be beneficial to determine whether the use of dopaminergic treatment might be effective in the treatment of post-traumatic depression.

## Highlights

• [^11^C]PHNO PET is used for the first time in traumatic brain injury (TBI) patients.
• Post-traumatic changes in dopamine D2/D3 receptors were observed.
• Patients with major depression showed more prominent reductions in [^11^C]PHNO BP_ND_.
• Non-depressed TBI patients had greater [^11^C]PHNO BP_ND_ in the Amygdala.
• These findings suggest a potential role of D2/D3 changes in post-TBI depression.

## 1 Introduction

Traumatic brain injury (TBI) often leads to cognitive and psychiatric problems (Whitnall et al., 2006; Fleminger and Ponsford, 2005). These are associated with dopaminergic abnormalities, including direct damage to dopaminergic nuclei, axonal injury to dopaminergic neurons and subsequent changes in dopamine production, metabolism and clearance (Jenkins et al., 2016; Bales et al., 2009). Neuroimaging studies of the human dopaminergic system after TBI have been limited. Striatal dopamine transporter (DAT) levels can be reduced (Wagner et al., 2005; Donnemiller et al., 2000; Wagner et al., 2014), with associated damage within the substantia nigra (SN) and nigrostriatal projections (Jenkins et al., 2018). Previous imaging studies have only investigated D2 receptors, with variable results (Donnemiller et al., 2000, Wagner et al., 2014). [^11^C]PHNO PET allows D2 and D3 receptors to be measured *in vivo* (Searle et al., 2010; Tziortzi et al., 2011), providing a more detailed characterization of post-traumatic dopamine receptor abnormalities.

Changes in dopaminergic function may be relevant to post-traumatic depression, which is very common (Jorge et al., 1993). Around a third of TBI patients develop major depressive disorder (MDD), with the risk increasing with greater injury severity (Kreutzer et al., 2001). Depression often hinders recovery and leads to long-term morbidity (Dikmen et al., 2003), but biological causes of MDD after TBI have seldom been investigated. In studies of non-traumatic MDD, alterations in dopamine receptors have been shown to be related to depressive symptoms (D'Haenen and Bossuyt, 1994) and abnormalities within the limbic system have often been observed (Drevets et al., 2008). High levels of D3 receptors are seen in limbic structures (Murray et al., 1994) and potentially play an important role in the development of neuropsychiatric disorders (Sokoloff et al., 2006). Hence, [^11^C]PHNO has the potential to clarify the aetiology of post-traumatic depression and inform a more targeted approach to treating this disabling condition.

Here we used [^11^C]PHNO PET to measure D2/D3 receptor availability following TBI. We tested the hypothesis that TBI patients would show abnormalities of [^11^C]PHNO binding and that patients with and without MDD will show distinct binding patterns. We used structural MRI to examine grey matter volume changes using voxel-based morphometry (VBM) to examine the relationship to D2/D3 receptor availability and diffusion tensor imaging (DTI) to investigate axonal injury and its relationship to D2/D3 receptor availability.

## 2 Material and methods

### 2.1 Study design and participants

Twelve TBI patients with a single moderate-severe TBI, as classified by the Mayo criteria (Malec et al., 2007), were assessed using [^11^C]PHNO PET, structural T1 MRI, DTI and neuropsychological tests in this cross-sectional study. A comparative group of 26 age-matched healthy controls were also assessed using [^11^C]PHNO PET and structural T1 MRI. For comparison of DTI, grey matter (GM) volume and cognitive performance, a second age-matched control group were recruited (*n* = 32). Demographics of healthy controls can be found in appendix Table B.1. Patients and controls were carefully selected to avoid the potential confound of medications. Specifically, individuals were not on any medication known to affect dopaminergic function which may confound [^11^C]PHNO BP_ND_. Patients classified with MDD (TBI-MDD) had no history of depression prior to their injury and had no other current DSM-IV diagnoses. All healthy controls and TBI patients classified as having no history of depression (TBI-NON) had no current or previous DSM-IV diagnoses.

### 2.2 Standard protocol approvals, registrations, and patient consents

This study was approved by the Westminster Research ethics committee. All participants gave written informed consent.

### 2.3 Neuropsychology

A neuropsychological test battery previously described to assess cognitive impairment after TBI (Kinnunen et al., 2011) was performed on patients and a control group. Tests included measures of processing speed, executive function, pre-morbid intellectual ability and memory.

### 2.4 Psychiatric assessment

TBI patients were classified as having MDD or no depressive symptoms based on the Structured Clinical Interview for DSM-IV-TR AXIS I Disorders, Version I/NP (SCID; overseen by a qualified psychiatrist, VR) (First et al., 2002). No other psychiatric illnesses were identified in any of the patients. In addition, patients completed the Beck Depression Inventory (BDI) (Beck and Beamesderfer, 1974) to assess current severity of major depressive symptoms.

### 2.5 Imaging protocol

Patients and controls underwent a single PET scan acquired using a Siemens Biograph 16 HI-REZ PET-CT scanner (Siemens Healthcare). The radiotracer [^11^C]PHNO was administered as an intravenous bolus over 30 s and dynamic PET data was acquired for 90 min. Injected [^11^C]PHNO did not exceed 3 μg total mass to avoid symptoms of nausea that have previously been reported. Administration of [^11^C]PHNO below 3 μg have previously been used to effectively quantify D2/D3 receptors *in vivo* (Erritzoe et al., 2014). A low-dose computed tomography (CT) image was acquired prior to administration of the radiotracer for attenuation correction during PET reconstruction. Further information about reconstruction of the dynamic PET images can be found in previously published literature (Searle et al., 2010). In summary, the PET data were binned into 26 frames (8 × 15 s, 3 × 60 s, 5 × 2 min, 5 × 5 min, 5 × 10 min) and reconstructed using Fourier re-binning and two-dimensional filtered back projection with a ramp filter at Nyquist cutoff frequency. Image data were smoothed with a Gaussian filter (5 mm full-width at half-maximum). Patients and controls who had PET scanning had a standard high-resolution T1 MPRAGE scan (160 1-mm-thick transverse slices, TR = 2300 ms, TE = 2.98 ms, FA = 9°, in-plane resolution = 1 × 1 mm, matrix size = 256 × 256, field of view = 25.6 × 25.6 cm), acquired for use in the co-registration of parametric [^11^C]PHNO BP_ND_ images. Standard high-resolution T1 was acquired using two Siemens 3T Verio MRI's (Siemens Healthcare).

For GM volume and DTI analyses, patients and a separate control group were scanned using the same Siemens 3T Verio MRI (Appendix fig. A.1). Acquisition of diffusion weighted imaging for diffusion tensor imaging analysis was performed on a 3T Veiro MRI using a 64-direction protocol with b = 1000 s/mm^2^ and four interleaved b = 0 s/mm^2^, with TE/TR 103/9500 ms, 64 contiguous slices, FoV 256 mm and voxel size 2 mm^3^. Susceptibility-weighted imaging (SWI) and fluid-attenuated inversion recovery (FLAIR) to facilitate assessment of focal lesions in patients was also acquired. No MRI analysis was performed across the two scanners.

### 2.6 Imaging analysis

Non-displaceable binding potential (BP_ND_) images of [^11^C]PHNO were generated using a molecular imaging and kinetic analysis toolbox (MIAKAT) (Gunn et al., 2016). The simplified reference tissue model (SRTM) (Lammertsma and Hume, 1996) with cerebellar grey matter (GM) as the reference region was used for generation of parametric D2/D3 BP_ND_ images, as per previous research (Searle et al., 2010).

T1 images were segmented into GM and white matter (WM) using SPM8. Tissue segmentations were then warped to a group template using a diffeomorphic non-linear image registration procedure (DARTEL) (Ashburner, 2007). The group template was then registered to Montreal Neurological Institute (MNI152) space using an affine registration. Parametric [^11^C]PHNO BP_ND_ images were co-registered to their relative T1 images using a rigid-body registration and then warped into MNI152 space using the template registration flow-fields obtained from the DARTEL registration. Normalized BP_ND_ images were then masked using a thresholded GM template and smoothed (8 mm full-width at half-maximum). To investigate the presence of GM atrophy, GM tissue segmentations of patients and controls acquired on the same scanner were warped to a group template using DARTEL and standardized to MNI152 space for use in voxel-based morphometry (VBM) (Ashburner and Friston, 2000).

Two types of analysis were performed to investigate [^11^C]PHNO BP_ND_: voxelwise analysis in images registered into standard MNI152 space and region-of-interest (ROI) analysis performed in subject T1 space. [^11^C]PHNO BP_ND_ values were extracted using an anatomical atlas previously developed for use in regional analysis of [^11^C]PHNO data (Tziortzi et al., 2011). The atlas was first affine-registered from MNI152 space to the group template. Next, the atlas was inversely warped to each subject's native T1 using DARTEL and subject-specific flow-fields. Native-space anatomical maps were visually assessed to ensure accuracy of registration. To further improve accuracy, ROIs were intersected with the subject's 30% thresholded GM tissue probability map (*p* > .3) to extract mean regional BP_ND_ values. Eight ROIs were selected *a priori* based on their involvement in the dopaminergic system, prominence of dopamine D2/D3 receptors and involvement in cognitive and emotional processing. The ROIs selected were; accumbens, amygdala, caudate, hypothalamus, pallidum, putamen, thalamus and substantia nigra. Although the ventral tegmental area (VTA) plays an important role in dopaminergic functioning within the brain, the size of this region and difficulty to accurately delineate the VTA could lead to unreliable results. We therefore did not explore this region in this analysis given the relatively low spatial resolution of PET and potential partial volume effects. Lesions present on T1 and FLAIR sequences in patients were manually segmented and excluded from ROI and voxelwise analysis.

Diffusion tensor imaging was used to derive FA as a measure of white matter microstructure. Fractional anisotropy maps were constructed using the DTI-TK toolbox (Zhang et al., 2007) and subsequently skeletonized for ROI analysis using TBSS (Smith et al., 2006). The mean FA for each ROI was extracted across patients and controls to investigate group differences and any relationship to [^11^C]PHNO BP_ND_. To examine the effect of TBI on dopaminergic neurons, the nigrostriatal projections (nigro-caudate, nigro-putamen, nigro-accumbens) were included in our analyses. A map of the area through which the nigrostriatal tract passes, as well as nigro-caudate, nigro-putamen and nigro-nucleus accumbens components of this map, was created using 100 subjects from the Human Connectome Project (HCP) (Van Essen et al., 2013). Fiber-tracking was performed in both directions between the left and right substantia nigra and the corresponding nucleus accumbens, putamen and caudate using the MRtrix package (Tournier et al., 2012). Three thousand tracks were performed with maximum angle between successive steps limited to 45°. A probability mask based on the percentage of tracks passing through each voxel was created for each individual and thresholded at 5%. All masks were binarised and masks from the two directions of tracking were combined. A group probabilistic mask that was then thresholded at 25% to create a group mask.

The FA values for controls and patients were then extracted using these tract masks to assess any relationships between white matter integrity and binding potentials. We also investigated the impact of TBI upon limbic connections using two tracts (the cingulum and uncinate fasciculus) from the Johns Hopkins University (JHU) WM atlas. These tracts were selected as they have previously been implicated in non-traumatic MDD (Zhang et al., 2012). Finally, we measured FA from the anterior thalamic projection as a control region which is commonly affected after injury.

### 2.7 Statistical analysis

Statistical analysis was performed in two stages. Firstly, all TBI patients were compared to controls to identify TBI-related differences. Secondly, to investigate the relationship between TBI and the presence of MDD, TBI patients were subdivided into TBI-MDD and TBI-NON groups and compared with controls.

#### 2.7.1 TBI patients and controls

Neuropsychological test performance between TBI patients and controls was assessed using independent samples *t*-tests and Mann Whitney-*U* tests using statistical software R, version 3.2.3. Voxelwise differences in BP_ND_ between patients and controls were investigated using non-parametric permutation tests in FSL using 10,000 permutations and including age and gender as covariates. Results were cluster corrected using threshold-free cluster enhancement and corrected for multiple comparison using family-wise error correction (*p* < .05). A repeated measures ANOVA was performed for ROI analysis of BP_ND_ across groups, including a group-by-ROI interaction term. Complementary pairwise comparisons with Bonferroni correction or one-way ANOVAs were performed to determine the drivers of any significant group-by-ROI interactions, again using R version 3.2.3. Previous literature has demonstrated age-related differences in dopaminergic levels (Kaasinen and Rinne, 2002) as well as time-dependent changes to dopaminergic function after TBI (Wagner et al., 2005). Therefore, Pearson's correlation was used to determine if there was a relationship between age, time since injury and regional [^11^C]PHNO BP_ND_.

GM volume differences between patients and controls were assessed using voxel-based morphometry (VBM). VBM was performed using the standardized GM tissue segmentations, using non-parametric permutation tests in FSL with 10,000 permutations. Age, gender and intracranial volume (ICV) were covariates in the analysis. Previous research has demonstrated reductions to SN volume after TBI and its relationship to dopaminergic changes (Jenkins et al., 2016). We therefore also examined SN volume and it's relationship to [^11^C]PHNO BP_ND_. GM volume was measured in the SN of patients and controls by intersecting a SN mask with standardized GM tissue segmentations. An independent samples *t*-test was used to examine group differences, corrected for age and ICV. SN volume was also correlated to [^11^C]PHNO BP_ND_ across the eight ROIs using Pearson's correlation to investigate any relationship.

#### 2.7.2 Comparison of MDD, non-MDD and healthy controls

Further investigation into the role of TBI in the development of MDD was assessed by comparing data across three groups (TBI-MDD, TBI-NON and healthy controls). Repeated measures ANOVA were performed across the three groups to examine group-by-ROI interactions. Subsequent one-way ANOVAs were performed to assess the group differences across regions.

## 3 Results

Twelve TBI patients (mean age 36.9 years, SD 9.9) were recruited (Table 1). Six patients were classified as having MDD (mean age 39.5 years, SD 11.2) and six as non-MDD (mean age 34.7 years, SD 9.7). For comparison of PET, a group of 26 healthy controls (mean age 35.9 years, SD 6.9) were included and a second group of 32 healthy controls (mean age 37.6 years, SD 9.1) were included for comparison of DTI, GM atrophy and neuropsychological measures.

**Table 1:** Demographics and clinical data of TBI patients.

Age	Sex	Education level	Classification	Mechanism of injury	PTA (days)	Medication	Time since injury (months)	Focal lesions	Microbleeds
53	M	University graduate	TBI-MDD	RTA	14	Citalopram 20 mg OD	268	Yes-Frontal	No
43	F	University graduate	TBI-MDD	RTA	3	Nil	47	No	Yes: frontal & parietal
22	M	School graduate	TBI-MDD	RTA	6	Nil	25	Yes-Frontal	No
49	F	University graduate	TBI-MDD	Fall	2	Sertraline 50 mg OD	17	Yes-Temporal	No
39	M	University graduate	TBI-MDD	NK	14	Ramipril 2.5 mg OD	23	No	No
32	M	University graduate	TBI-MDD	Fall	1	Nil	41	No	Yes-Temporal
40	M	University graduate	TBI-NON	RTA	42	Nil	125	No	No
32	M	University graduate	TBI-NON	RTA	90	Nil	55	No	Yes-Frontal
40	F	University graduate	TBI-NON	Fall	3	Nil	52	No	No
25	M	University graduate	TBI-NON	RTA	14	Nil	15	Yes-Temporal	Yes-lentiform nucleus (subcortical)
23	F	University Graduate	TBI-NON	Fall	2	Nil	28	Yes-Frontal	No
48	M	School Graduate	TBI-NON	RTA	60	Nil	372	Yes-Temporal	Yes-Temporal

Table of patient demographics and psychiatric classification (MDD = Major depressive disorder, NON-MDD = no classification of major depressive disorder). PTA = Post-traumatic amnesia. RTA = Road traffic accident. OD = once daily.

A consultant neuroradiologist reviewed the structural MRI scans. Six TBI patients had no focal lesions as defined on T1 and FLAIR. The remaining six patients had focal lesions in the frontal (*n* = 3) and temporal (*n* = 3) lobes (Fig. A.2). No focal lesions were present in subcortical areas. Microbleeds as defined on SWI, were present in five patients. Of these five patients, one was found to have microbleeds subcortically in the lentiform nucleus (Table 1).

TBI patients showed impairments on a number of neuropsychological tests compared to age-matched controls. Performance was significantly poorer on tasks involving processing speed and executive function (Table 2). There were no differences in pre-morbid intellectual ability across the two groups. Performance on neuropsychological tests did not differ between TBI-MDD and TBI-NON patients.

**Table 2:** Neuropsychology performance for TBI patients and controls.

Neuropsychological test	Controls (*n* = 32)	Patients (*n* = 12)	*p*
Processing speed			
Stroop colour naming	27.56 ± 4.72	34.18 ± 8.55	**.015**⁎
Stroop word reading	20.44 ± 4.48	26.64 ± 8.05	**.016**⁎
Trail making A	19.62 ± 7.13	30.27 ± 17.04	**.034**⁎

Executive function			
Trail making B	44.84 ± 22.67	69.82 ± 37.90	**.030**⁎
Stroop inhibition	49.97 ± 11.63	50.36 ± 11.78	.462
Stroop inhibition switching	55.59 ± 15.42	66.0 ± 22.14	.078
Trail making test B-A	25.28 ± 19.02	39.55 ± 25.35	**.046**⁎

Memory			
WMS-III LM1 Immediate recall	50.25 ± 8.44	48.18 ± 11.16	.292
WMS-III LM2 delayed recall	32.38 ± 7.024	33.5 ± 8.49	.645
Peoples test immediate recall	28.44 ± 5.75	27.18 ± 9.83	.348
Peoples test delayed recall	10.12 ± 2.90	9.64 ± 2.73	.310

Intellectual ability			
WTAR scaled score	114.9 ± 8.30	107.3 ± 19.91	.240
WASI matrix reasoning	28.88 ± 4.98	28.73 ± 3.20	.910

Mean and SD of neuropsychological test scores for patients and controls are presented. WMS-II=Wechsler memory scale, second edition. LM = Logical memory. WTAR = Wechsler adult test of reading, WASI=Wechsler abbreviated scale of intelligence.

⁎ *p* < .05.

### 3.1 TBI is associated with reduced caudate D2/D3 receptor availability

**Fig. 1:**
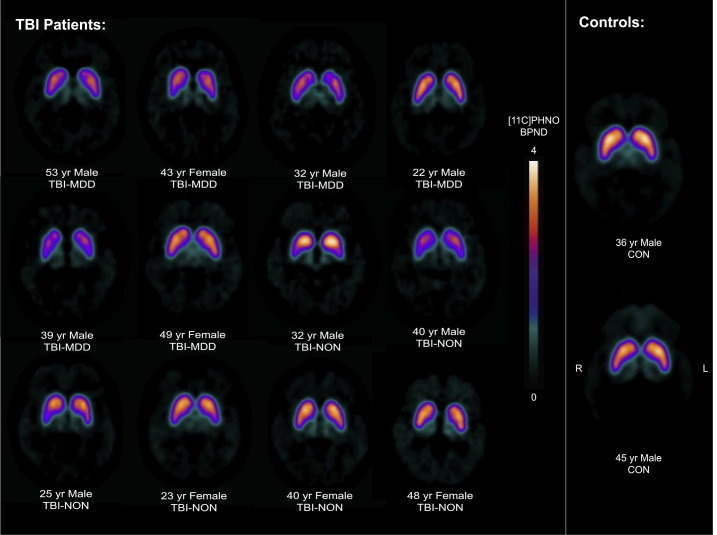
[^11^C]PHNO PET BP_ND_ images of TBI patients and two representative healthy controls. Figure illustrates parametric [^11^C]PHNO BP_ND_ images for TBI patients and two healthy controls (CON). Scans are annotated for patients with (TBI-MDD) and without depression (TBI-NON) and age. [^11^C]PHNO BP_ND_ is shown in the colour bar. (For interpretation of the references to color in this figure legend, the reader is referred to the web version of this article.)

**Fig. 2:**
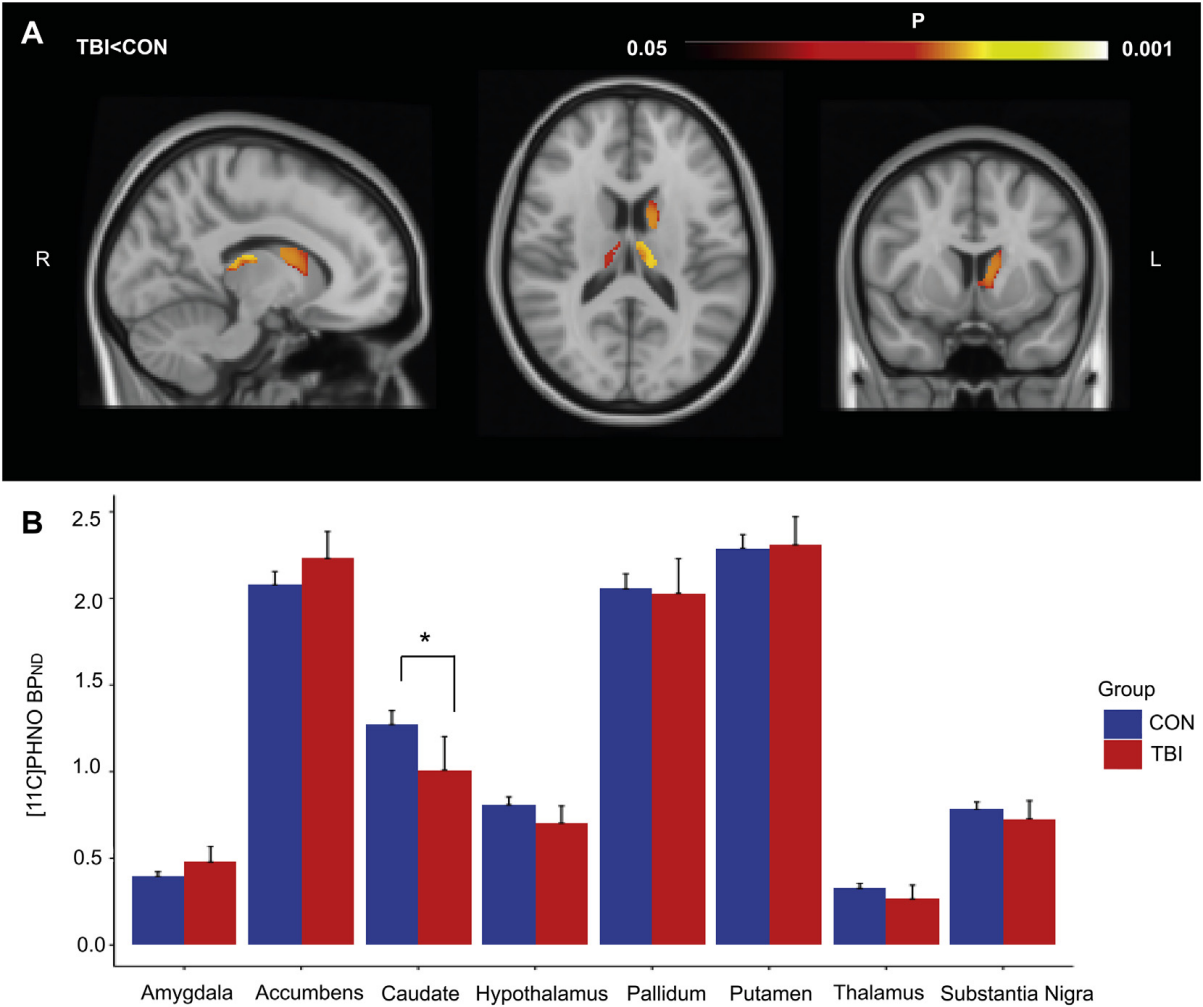
Comparison of [^11^C]PHNO BP_ND_ between TBI patients and controls. Results of the two statistical comparisons of [^11^C]PHNO BP_ND_ between TBI patients (PAT) and controls (CON). (A) Voxelwise results showing areas of significant reduction in [^11^C]PHNO BP_ND_ in patients compared to controls (*p* < .05). (B) Region-of-interest analysis of BP_ND_ between TBI patients and controls. Error bars represent 95% confidence interval. ^⁎^*p* < .05 (Bonferroni corrected).

Individual [^11^C]PHNO BP_ND_ images are shown in Fig. 1. As expected, [^11^C]PHNO BP_ND_ was concentrated in D2/D3 rich structures such as the striatum, although there was a patchy reduction apparent in many patients. Voxelwise comparison of [^11^C]PHNO BP_ND_ revealed areas of lower BP_ND_ in the caudate and thalamus of TBI patients (Fig. 2A.). No regions showed increased BP_ND_ in TBI patients compared to controls.

A confirmatory ROI analysis was performed. Repeated measures ANOVA of BP_ND_ across the regions showed a significant group-by-region interaction (F(7,245) = 6.4, *p* = .001), Greenhouse-Geisser corrected. This interaction resulted from a specific reduction of BP_ND_ in the caudate for TBI patients (*p* = .025) (Fig. 2B). Age and time since injury did not correlate with regional BP_ND_.

### 3.2 Major depressive disorder (MDD) following TBI is associated with altered D2/D3 receptor availability

**Fig. 3:**
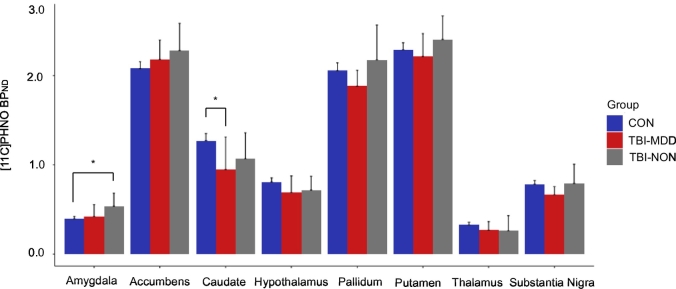
Region-of-interest [^11^C]PHNO BP_ND_ between healthy controls and patients with and without MDD. Results of region-of-interest analysis of [^11^C]PHNO BP_ND_ between healthy controls (CON) and TBI patients with (TBI-MDD) and without (TBI-NON) depression. Error bars represent 95% confidence intervals. ^⁎^*p* < .05.

We assessed the relationship between [^11^C]PHNO binding and MDD using a ROI approach (Fig. 3). Repeated measures ANOVA across the three groups (TBI-MDD, TBI-NON and controls) showed a significant group-by-region interaction (F(14,238) = 4.104, *p* = .001), Greenhouse-Geisser corrected. Complementary one-way ANOVAs revealed that this interaction was the result of a significant main effect of group in the caudate (F(2,34) = 5.398, *p* = .009), with post-hoc *t*-tests indicating significantly reduced binding in the caudate of TBI-MDD patients compared to controls (*p* = .014). A significant effect of group was also present in the amygdala (F(2,34) = 5.81, *p* = .007), with post-hoc t-tests revealing increased binding for TBI-NON patients compared to controls (*p* = .005). There were no significant differences in [^11^C]PHNO BP_ND_ between the two TBI patient groups. Age and time since injury did not correlate with regional BP_ND_ across the three groups. TBI-MDD patients had significantly higher BDI scores (mean = 21.83), U = 29, *p* = .045, than TBI-NON patients (mean = 7.17). However, there was no correlation between BDI scores and BP_ND_ in any region.

### 3.3 Control analyses for positron emission tomography results

As expected, patients had reduced GM volume in the frontal lobes, striatum and cerebellum. As cerebellar GM is used as the reference tissue region for modeling [^11^C]PHNO BP_ND_ this might potentially confound our analysis. However, there were no group differences in the standard uptake values (SUVs) in the cerebellum used in the calculation of parametric [^11^C]PHNO BP_ND_ images between patients and controls (t(34.557) = 0.852, *p* = .399) or across the three groups (F(2,35) = 0.637, *p* = .535) (Fig. A.3). Reduced GM volume observed in the striatum of patients may contribute to reduced [^11^C]PHNO uptake. However, linear regression revealed no relationship between GM volume and [^11^C]PHNO BP_ND_ in regions within the striatum of patients or in any other ROI's included in our analysis (*p* > .3).

### 3.4 Nigro-caudate tract fractional anisotropy correlates with D2/D3 receptor availability in the caudate

**Fig. 4:**
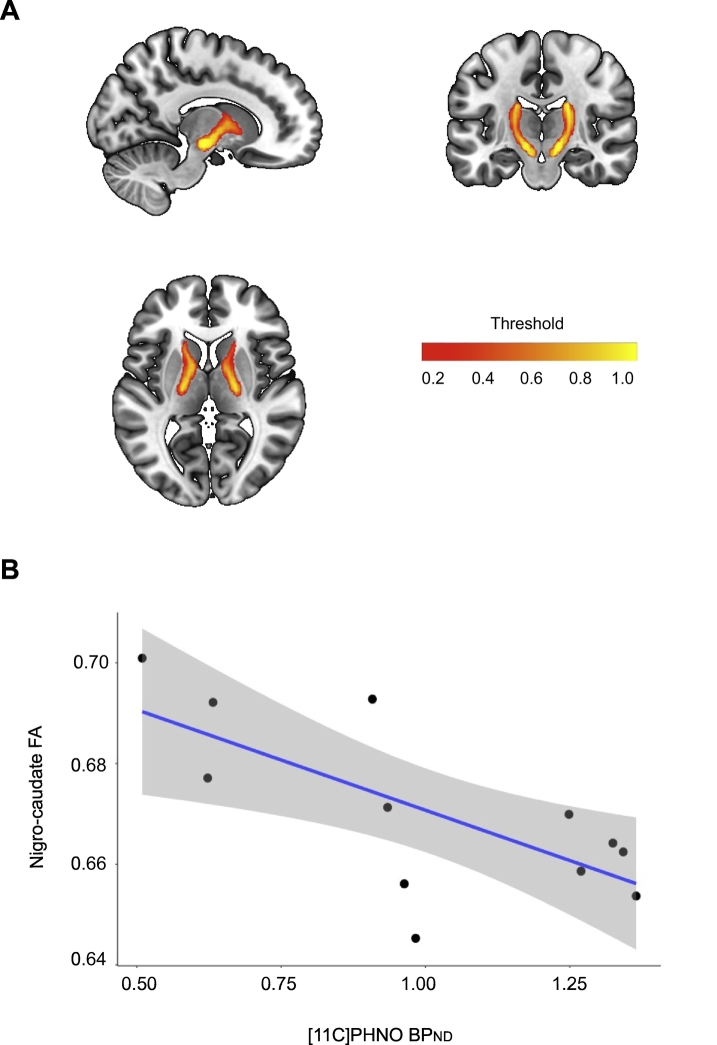
Nigro-caudate tract fractional anisotropy and caudate [^11^C]PHNO BP_ND_ in TBI patients. (A) Probabilistic map of nigro-caudate tract derived from tractography of 100 healthy controls from the human connectome project. Colour bar showing fractional anisotropy (FA) (B) Relationship between nigro-caudate tract FA and caudate [^11^C]PHNO BP_ND_ in TBI patients.

We have previously shown relationships between diffusion MRI measures in white matter through which the nigrostriatal pathways pass and dopamine transporter imaging (DAT) (Jenkins et al., 2018). Therefore, to explore the reasons for abnormal [^11^C]PHNO BP_ND_ we investigated their relationship to these diffusion metrics. Within TBI patients, linear regression revealed a significant relationship between nigro-caudate tract FA and [^11^C]PHNO BP_ND_ in the caudate (b = −0.04, SE = 0.01, *t* = −3.12, *p* = .01), such that greater FA related to lower [^11^C]PHNO caudate binding (Fig. 4). There was no relationship between [^11^C]PHNO BP_ND_ in the accumbens or putamen and FA within the projections to these structures from the substantia nigra. Despite these relationships there were no significant differences in FA between controls and patients in the three nigrostriatal pathways.

### 3.5 Substantia Nigra volume does not correlate with D2/D3 receptor availability in patients

We also investigated whether reductions to dopaminergic nuclei volume were present in patients and whether this related to reduced [^11^C]PHNO BP_ND_ in the caudate of TBI patients. There was no significant difference between TBI patients and controls for SN volume, corrected for age and ICV, although this approached significance (*p* = .065). We did not find a relationship between SN volume and caudate [^11^C]PHNO BP_ND_ in patients.

### 3.6 TBI patients with major depressive disorder have significantly reduced FA in white matter tracts that connect the limbic system

**Fig. 5:**
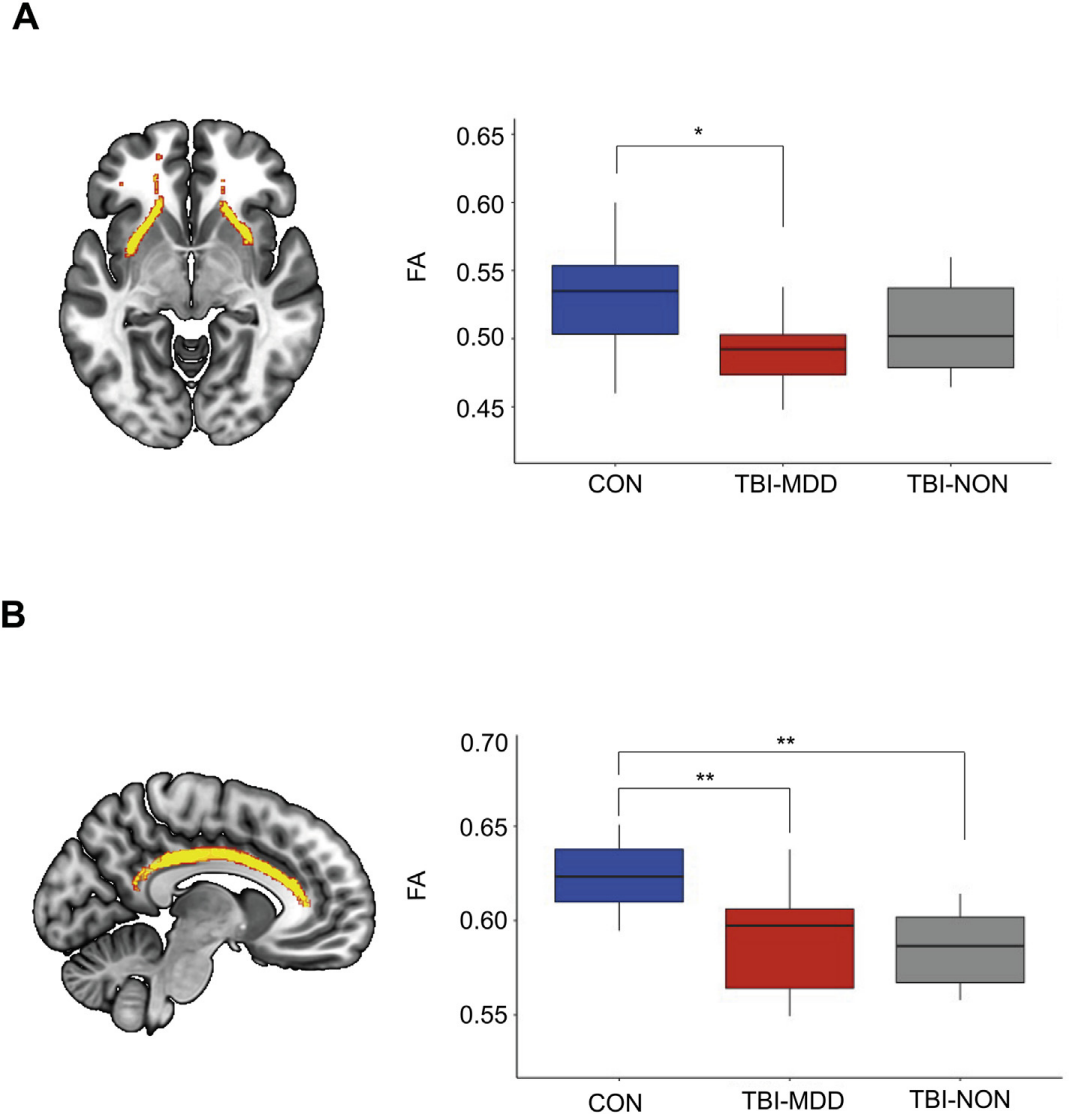
Fractional anisotropy in limbic tracts across patients and controls. (A) Results of regional FA analysis in the uncinate fasciculus across TBI patients classified with (TBI-MDD) or without (TBI-NON) depression and healthy controls (CON). (B) Results of regional FA analysis in the cingulum across the three groups. Tracts are represented in yellow in brain images. ^⁎^*p* < .05, ^⁎⁎^*p* < .01.

We next investigated whether TBI patients with MDD have greater evidence of axonal injury compared to TBI-NON patients and controls. One-way ANOVAs performed across the three groups (TBI-MDD, TBI-NON and controls) revealed a significant main effect of group for the cingulum (F(2,39) = 10.83, *p* < .001) which was driven by significantly reduced FA in TBI-MDD patients (*p* = .007) and TBI-NON patients (*p* = .001) compared to controls. There was also a main effect of group in the uncinate fasiculus (F(2.39) = 4.121, *p* = .02), driven by significantly reduced FA in TBI-MDD patients compared to controls (*p* = .03). There were no significant differences between the two patient groups (Fig. 5).

## 4 Discussion

Here we present the first use of [^11^C]PHNO PET to investigate dopaminergic abnormalities following TBI. We show that [^11^C]PHNO BP_ND_ is lower in the caudate after TBI, and that this abnormality is more prominent in patients with MDD. In addition, greater binding was seen in non-depressed patients in the amygdala compared to controls, suggesting that compensatory changes in the dopamine system might be protective against the development of MDD after TBI.

[^11^C]PHNO binding reflects the availability of D2 and D3 receptors and can be attributed to receptor density as well as endogenous dopamine levels (Schotte et al., 1996). Although [^11^C]PHNO preferentially binds to D3 receptors with a 20 fold selectivity over D2 receptors (Gallezot et al., 2012), binding in regions containing both receptors will represent mixed D2 and D3 signal. The relative contributions of D2 and D3 receptors to [^11^C]PHNO signal within a region is an important consideration when interpreting our findings. Blockade studies using D3-specific antagonists have illustrated that D2 receptors are predominantly expressed in the caudate (Searle et al., 2010; Tziortzi et al., 2011). Therefore, reductions in caudate binding seen in TBI patients may primarily reflect reduced availability of D2 receptors after TBI. In contrast, the amygdala consists of a mixture of D2 and D3 receptors, as shown by human post-mortem studies (Murray et al., 1994; Gurevich and Joyce, 1999). Given the high affinity of [^11^C]PHNO for D3 receptors, increased binding within the amygdala is likely to indicate increased expression of D3 receptors in this region. Baseline and blockade scans using a D3-specific antagonist to quantify the relative D2 and D3 contributions to [^11^C]PHNO signal in the amygdala would help to further disentangle the effects of TBI on D2 and D3 receptors in our patients.

Our findings extend previous work demonstrating dopaminergic abnormalities after TBI (Donnemiller et al., 2000; Wagner et al., 2014). Animal models of TBI show that dopaminergic abnormalities are common and the behavioural consequences can be treated with dopamine re-uptake inhibitors (Wagner et al., 2009). In humans, TBI frequently disrupts dopaminergic pathways through a variety of mechanisms. Focal brainstem damage can directly damage brainstem dopaminergic nuclei, and traumatic axonal injury can damage their white matter projections (Jenkins et al., 2018). The effect of these changes has consistently been observed as a reduction in DAT in the striatum after TBI (Wagner et al., 2005; Donnemiller et al., 2000; Wagner et al., 2014). DAT is the main regulator of synaptic dopamine levels and this change is likely to be due to a compensatory down-regulation in response to reduced synaptic dopamine levels. Although we did not find evidence of axonal injury in dopaminergic projections, nigro-caudate FA was related to dopaminergic abnormalities in the caudate of TBI patients, suggesting an important role for dopaminergic projections and the compensatory changes to the dopaminergic pathways observed after TBI.

A small number of studies on the effects of TBI on dopamine receptor distributions have been conducted but D3 receptors have not previously been investigated. Animal studies have not provided evidence for chronic changes in D2 receptors after TBI (Wagner et al., 2005). However in humans, chronic changes have been reported. Donnemiller and colleagues reported reductions of striatal D2 receptor binding using the SPECT tracer ^123^I-IBZM in patients who also had reductions of DAT in this region (Donnemiller et al., 2000). In contrast, higher D2 receptor binding has been seen in the ventral striatum using the PET ligand [^11^C]raclopride (Wagner et al., 2014). Our results replicate the finding of reduced D2 receptor binding in the caudate, but also provide some evidence of variability in the effect of TBI on dopamine receptors as [^11^C]PHNO binding was increased in patients without MDD in the amygdala. These results suggest multiple factors may influence levels of D2 and D3 receptors following TBI which require further exploration.

Compensatory reductions in DAT could lead to a direct reduction in post-synaptic D2 receptors, whereas increased binding in the amygdala might be due to a compensatory upregulation of receptors in response to reduced synaptic dopamine levels. However, changes to endogenous dopamine levels might also contribute to the alterations in [^11^C]PHNO BP_ND_ seen in our patients without a direct relationship to receptor density changes. Reductions in DAT can increase synaptic dopamine levels following TBI. Increased synaptic dopamine levels may provide greater competition with [^11^C]PHNO to bind to D2/D3 receptors thus leading to decreased [^11^C]PHNO BP_ND_ within these regions. Extending the current work to include quantification of DAT in addition to [^11^C]PHNO BP_ND_ would provide a greater understanding of the mechanisms behind changes in [^11^C]PHNO BP_ND_.

Dopaminergic abnormalities caused by TBI may be related to post-traumatic depression. In keeping with this possibility, we observed patterns of [^11^C]PHNO binding in patients with and without post-traumatic depression, which suggest the potential involvement of dopaminergic abnormalities in post-traumatic depression. Abnormally low [^11^C]PHNO binding was only observed in TBI-MDD patients compared to controls when patients were grouped by psychiatric status, suggesting a potential role for altered dopamine function in the caudate in post-traumatic depression. In individuals with non-traumatic MDD, lower striatal DAT has been observed (Meyer et al., 2001) whilst increases in D2 receptor binding have been associated with recovery from clinical depression over time (Larisch et al., 1997). A relationship between dopaminergic abnormalities and depressive symptoms has also been observed in other neurological conditions. Depression is commonly seen in Parkinson's disease (PD), where striatal DAT and D2 receptor levels are often reduced (Vriend et al., 2014; Antonini et al., 1997). Depressed patients with PD have also been shown to have significantly reduced DAT binding in the caudate compared to non-depressed PD patients (Remy et al., 2005). Similarly, in Huntington's disease (HD) (a disease associated with neuronal cell loss in the caudate (Roth et al., 2005) and dopaminergic abnormalities), many patients develop depressive illness (Paulsen et al., 2005).

TBI patients without MDD showed higher [^11^C]PHNO binding in the amygdala compared to controls, which might indicate a compensatory change in D3 receptor expression that protects against the development of depression after TBI. However, this must be considered carefully given the small group sizes and no significant differences being detected between TBI-MDD and TBI-NON patient groups. Nevertheless, the amygdala is widely implicated in the regulation of mood and affect. In non-TBI depressed patients, pathophysiological changes to this region have included alterations in resting glucose metabolism, volume and dopamine transmission (Drevets, 2003; Klimek et al., 2002). Dopamine plays an important role in the modulation of the amygdala and its response to stimuli (Kienast et al., 2008). Interestingly, animal models have demonstrated that antidepressant treatments (such as tricyclic antidepressants) selectively increase D3 receptor expression (Lammers et al., 2000) and D3-receptor agonists such as pramipexole have antidepressant properties in both humans and animal models (Willner, 1983; Goldberg et al., 1999). It is conceivable that D3 receptor upregulation after TBI might provide similar benefits to antidepressant treatment and that measuring D2/D3 receptor levels might inform the choice of effective anti-depressant medication after TBI.

We also found evidence for abnormalities in white matter connections within the limbic system, with more widespread damage seen in depressed patients. Diffusion abnormalities were seen in the cingulum and uncinate fasciculi bilaterally. These white matter tracts connect nodes in the limbic system and similar diffusion abnormalities in these tracts have previously been observed in patients with non-traumatic depression (Zhang et al., 2012; Keedwell et al., 2012). Damage to the uncinate fasciculus connections were only seen in depressed TBI patients, suggesting that damage to the connections between the prefrontal cortex and amygdala/hippocampal gyrus may be an important factor in the development of post-traumatic depression.

There are a number of potential limitations to our work. Firstly, as expected, there was evidence of post-traumatic atrophy in some brain regions. It is possible that this might have influenced our PET results. However, this is unlikely to be a major influence on our results as there was no relationship between regional volume and binding in either the caudate or any other ROI. In addition, cerebellar SUVs were similar in the control and patient groups indicating that there was not a systematic bias between the groups in our reference regions. Secondly, we focused on studying D2/D3 dopaminergic receptors. It is possible to provide a more complete description of post-traumatic dopaminergic abnormalities and future research would benefit from measuring additional factors such as DAT levels in conjunction with [^11^C]PHNO binding. In addition, we used an automated atlas method to derive ROIs. This process may be susceptible to noise due to misalignment in smaller subcortical regions compared to manual delineation. However, we used an advanced DARTEL registration method that has previously been used for PET data and has superior registration to earlier non-linear techniques.

Our study had small sample sizes within our TBI-MDD and TBI-NON patient groups, which might have impacted on the power to detect differences. Hence, our findings should be viewed as preliminary evidence for the role of dopaminergic changes in TBI-MDD. We did not observe any significant differences between the two TBI groups but did identify different patterns of altered [^11^C]PHNO BP_ND_ when comparing each group to healthy controls. Future studies would benefit from larger group sizes to determine whether there are distinct differences between TBI-MDD and TBI-NON patients that we were underpowered to detect. This would help to further clarify whether the observed patterns of altered binding across the TBI groups are specific to psychiatric outcomes. Although the sample sizes are small between these groups, our findings suggest some potentially interesting D2/D3 regions to explore further in much larger patient groups.

## 5 Conclusions

In conclusion, we provide evidence of altered D2/D3 receptor binding after TBI, using [^11^C]PHNO PET for the first time in this patient population. Patterns of D2/D3 receptor abnormalities in clinically depressed and non-depressed TBI patients varied when compared to controls. Lower caudate binding supports previous findings of D2 abnormalities after TBI (Jenkins et al., 2018) and provide a potential mechanistic link between altered dopaminergic function in the caudate and the development of post-traumatic depression. These findings are preliminary, and future research extending this work using larger sample sizes is recommended.

## Appendix A Supplementary data

Supplementary figures and data Image 1
