# Supplementary material for: Dopamine D2/D3 receptor abnormalities after traumatic brain injury and their relationship to post-traumatic depression
Source: Neuroimage Clin. 2019 Jul 22;24:101950. doi: 10.1016/j.nicl.2019.101950 (PMC6664227; doi:10.1016/j.nicl.2019.101950)
Supplement: Supplementary file 1 — Supplementary figures and data [file mmc1.docx]

**Appendix A: Supplementary figures**


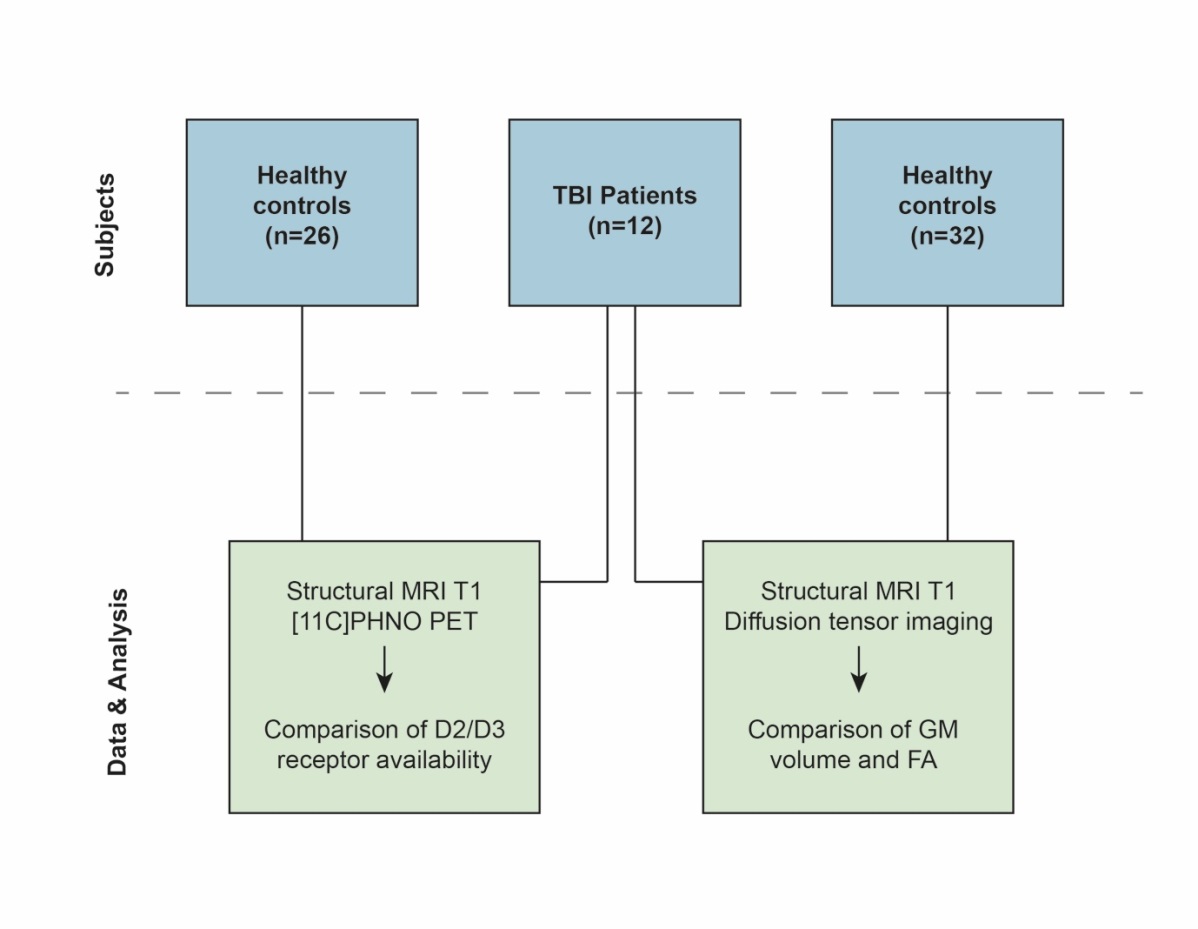


**Figure A.1: Schematic of subjects used, and analysis performed.** Schematic demonstrates which cohorts were used for each analysis performed. GM=Grey matter, FA=Fractional anisotropy.

**
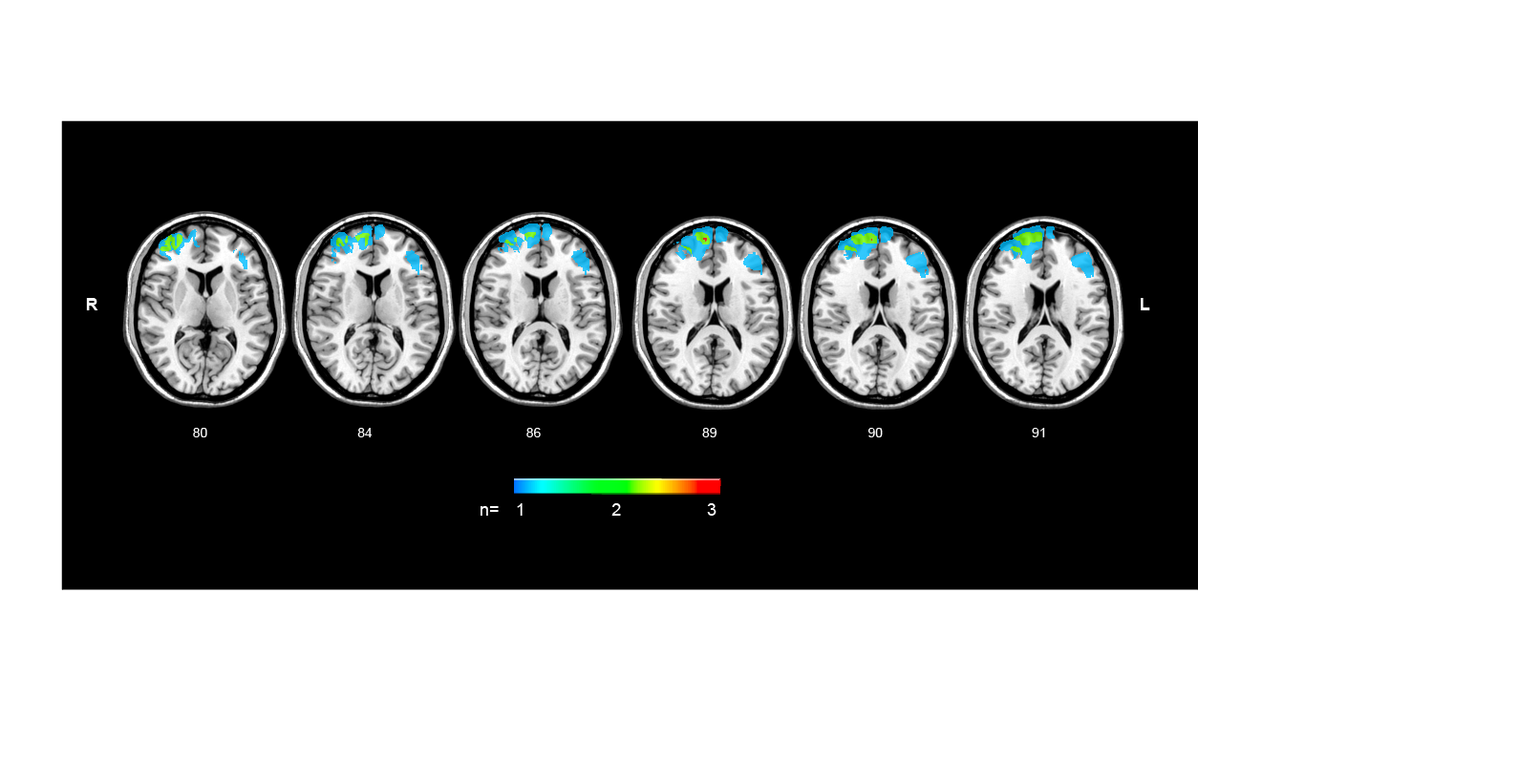
**

**Fig. A.2: Lesion overlap map for TBI patients with focal lesions**

Lesions were defined using FLAIR and T1 imaging. Colors represent the number of individuals with lesions in overlapping voxels.


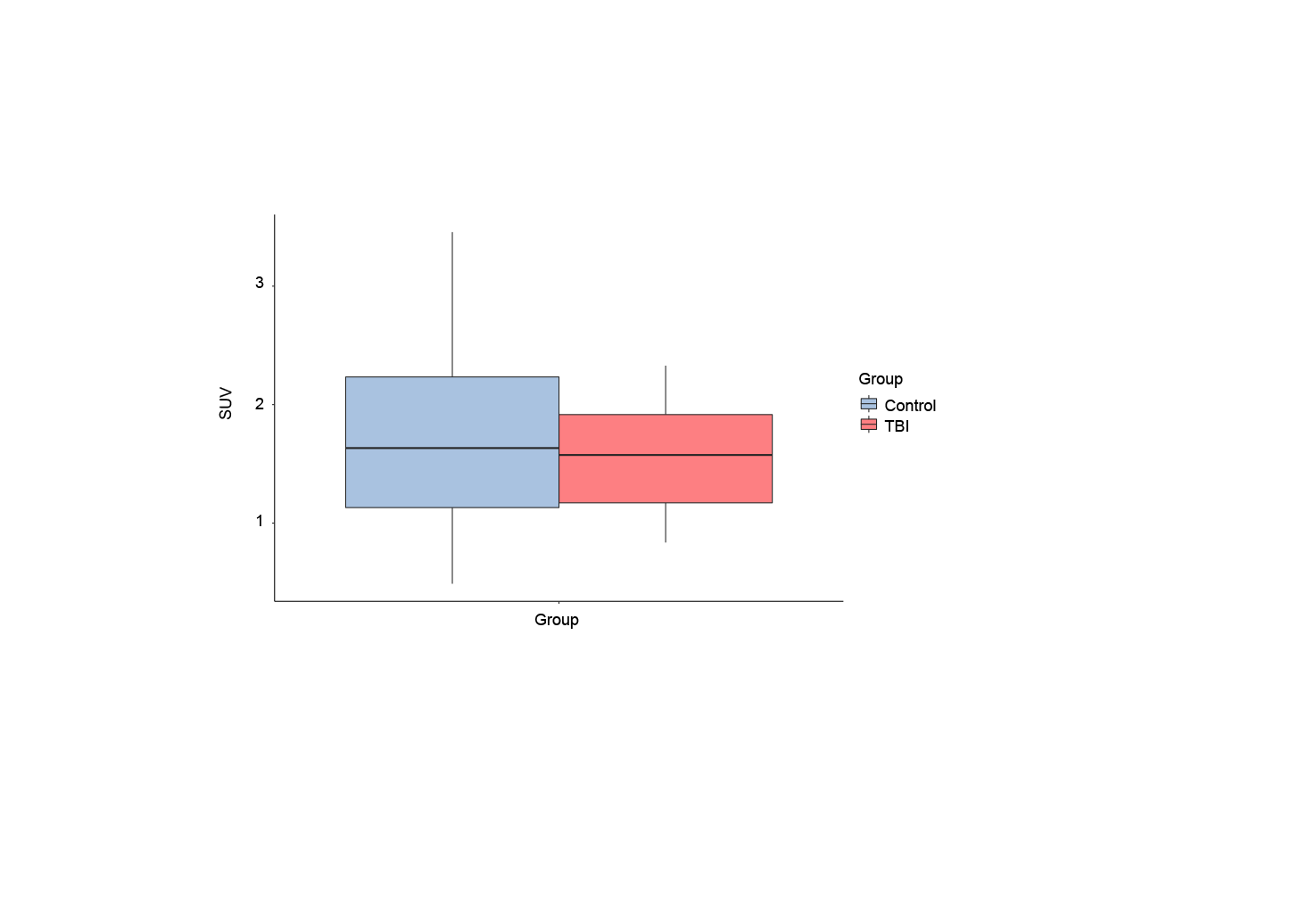


**Fig. A.3: [^11^C]PHNO Standard uptake values in the Cerebellum of TBI patients and controls.**

**Appendix B: Supplementary data**

| Age | Sex | Classification | Analysis |
| --- | --- | --- | --- |
| 21 | Female | Control | DTI /VBM |
| 42 | Male | Control | DTI /VBM |
| 21 | Male | Control | DTI /VBM |
| 43 | Female | Control | DTI /VBM |
| 49 | Male | Control | DTI /VBM |
| 44 | Male | Control | DTI /VBM |
| 45 | Female | Control | DTI /VBM |
| 29 | Male | Control | DTI /VBM |
| 34 | Male | Control | DTI /VBM |
| 35 | Male | Control | DTI /VBM |
| 46 | Male | Control | DTI /VBM |
| 47 | Male | Control | DTI /VBM |
| 33 | Male | Control | DTI /VBM |
| 25 | Male | Control | DTI /VBM |
| 35 | Female | Control | DTI /VBM |
| 24 | Male | Control | DTI /VBM |
| 35 | Male | Control | DTI /VBM |
| 36 | Male | Control | DTI /VBM |
| 47 | Male | Control | DTI /VBM |
| 56 | Male | Control | DTI /VBM |
| 28 | Male | Control | DTI /VBM |
| 36 | Male | Control | DTI /VBM |
| 41 | Male | Control | DTI /VBM |
| 39 | Male | Control | DTI /VBM |
| 49 | Male | Control | DTI /VBM |
| 35 | Male | Control | DTI /VBM |
| 39 | Male | Control | DTI /VBM |
| 34 | Male | Control | DTI /VBM |
| 30 | Male | Control | DTI /VBM |
| 45 | Male | Control | DTI /VBM |
| 36 | Male | Control | PET |
| 45 | Male | Control | PET |
| 34 | Male | Control | PET |
| 53 | Male | Control | PET |
| 32 | Male | Control | PET |
| 39 | Male | Control | PET |
| 37 | Male | Control | PET |
| 36 | Male | Control | PET |
| 40 | Male | Control | PET |
| 35 | Male | Control | PET |
| 36 | Male | Control | PET |
| 35 | Male | Control | PET |
| 31 | Male | Control | PET |
| 52 | Male | Control | PET |
| 33 | Male | Control | PET |
| 40 | Male | Control | PET |
| 42 | Male | Control | PET |
| 31 | Male | Control | PET |
| 40 | Male | Control | PET |
| 28 | Male | Control | PET |
| 29 | Male | Control | PET |
| 38 | Male | Control | PET |
| 28 | Male | Control | PET |
| 26 | Male | Control | PET |
| 30 | Male | Control | PET |

**Table B.1: Demographics of healthy controls used in analysis.** Analysis column represents which part of analysis healthy controls were used in. DTI/VBM=healthy controls compared with TBI patients for DTI and grey matter volume (VBM). PET=Healthy controls used in the comparison of [^11^C]PHNO BP_ND_ with TBI patients.
